# The Emergence and Spread of Multiple Livestock-Associated Clonal Complex 398 Methicillin-Resistant and Methicillin-Susceptible *Staphylococcus aureus* Strains among Animals and Humans in the Republic of Ireland, 2010–2014

**DOI:** 10.1371/journal.pone.0149396

**Published:** 2016-02-17

**Authors:** Gráinne I. Brennan, Yvonne Abbott, Aisling Burns, Finola Leonard, Brenda A. McManus, Brian O’Connell, David C. Coleman, Anna C. Shore

**Affiliations:** 1 National MRSA Reference Laboratory, St. James’s Hospital, James’s St., Dublin 8, Ireland; 2 Microbiology Research Unit, Division of Oral Biosciences, Dublin Dental University Hospital, University of Dublin, Trinity College Dublin, Dublin 2, Ireland; 3 Pathobiology, School of Veterinary Medicine, University College Dublin, Belfield, Dublin 4, Ireland; 4 Department of Clinical Microbiology, University of Dublin, Trinity College, St. James’s Hospital, James’s St., Dublin 8, Ireland; Amphia Ziekenhuis, NETHERLANDS

## Abstract

Clonal complex (CC) 398 methicillin-resistant *Staphylococcus aureus* (MRSA) and methicillin-susceptible *S*. *aureus* (MSSA) are associated with carriage and infection among animals and humans but only a single case of CC398 MRSA has been reported in the Republic of Ireland (ROI). The present study investigated the molecular epidemiology of CC398 MRSA (*n* = 22) and MSSA (*n* = 10) from animals and humans in the ROI from 2010–2014. Isolates underwent antimicrobial susceptibility testing, *spa* typing, DNA microarray profiling and PCR for CC398-associated resistance genes. All MRSA underwent SCC*mec* IV or V subtyping. Four distinct CC398-MRSA incidents were identified from (i) a man in a nursing home (*spa* type t011-SCC*mec* IVa, immune evasion complex (IEC) negative), (ii) a horse and veterinarian who had recently travelled to Belgium (t011-IVa, IEC positive), (iii) pigs (*n* = 9) and farm workers (*n* = 9) on two farms, one which had been restocked with German gilts and the other which was a finisher farm (t034-V_T_, IEC negative, 3/9 pigs; t011- V_T_, IEC negative, 6/9 pigs & 9/9 farm workers), and (iv) a child who had worked on a pig farm in the UK (t034-V_T_, IEC negative). Isolates also carried different combinations of multiple resistance genes including *erm*(A), *erm*(B), *tet*(K), *tet*(M) & *tet*(L), *fexA*, *spc*, *dfrG*, *dfrK aacA-aphD* and *aadD* further highlighting the presence of multiple CC398-MRSA strains. CC398 MSSA were recovered from pigs (*n* = 8) and humans (*n* = 2). CC398 MSSA transmission was identified among pigs but zoonotic transmission was not detected with animal and human isolates exhibiting clade-specific traits. This study highlights the importation and zoonotic spread of CC398 MRSA in the ROI and the spread of CC398 MSSA among pigs. Increased surveillance is warranted to prevent further CC398 MRSA importation and spread in a country that was considered CC398 MRSA free.

## Introduction

Clonal complex 398 (CC398) livestock-associated methicillin-resistant *Staphylococcus aureus* (LA-MRSA) was first reported in 2005 from pigs, pig handlers and their close contacts in the Netherlands [1]. Subsequently it was identified from a range of livestock and livestock-derived food products and horses as well as in humans, predominantly those with contact with livestock, in several countries, particularly in regions with high-density pig farming in continental Europe, Canada, Asia and the USA [2]. While CC398 MRSA is predominantly associated with animal colonisation, serious human infections as well as spread to and within the healthcare system have been reported [2]. Methicillin-susceptible *S*. *aureus* (MSSA) belonging to CC398 have also been reported from animals and humans and have been associated with community- and healthcare-associated infections in humans, many without livestock contacts [3–6]. Phylogenetic studies have identified human and LA CC398 clades and have revealed that LA CC398 MRSA emerged from human CC398 MSSA via acquisition of the staphylococcal cassette chromosome *mec* (SCC*mec*) element and tetracycline resistance genes *tet*(M) and loss of the phage-encoded immune evasion complex (IEC) genes [7, 8]. In human *S*. *aureus* strains (both MSSA and MRSA) the IEC genes are encoded in the genomes of a specific group of related lysogenic bacteriophages that integrate into and inactivate the *S*. *aureus* chromosomal beta-toxin gene *hlb* [9, 10]. Animal strains of *S*. *aureus* usually lack these bacteriophages and are IEC-negative.

Despite its prevalence in continental Europe and sporadic reports of CC398 MRSA in the UK (including Northern Ireland) among piglets, horses, turkeys, bovine bulk tank milk and retail pork [11–15], only a single case of CC398 MRSA has been reported in the Republic of Ireland (ROI), from an elderly man in a nursing home in 2012 and our pig population has remained CC398 free [16, 17]. Here we report molecular epidemiological evidence of the emergence and spread of CC398 MRSA and MSSA among animals and humans in the ROI, and evidence of the importation and zoonotic spread of CC398 MRSA.

## Materials and Methods

### Ethics statement

All isolates identified by the Irish National MRSA Reference Laboratory (NMRSARL) were collected as part of routine clinical care. The samples from the horse and the pig submitted for postmortem at the University College Dublin Veterinary Hospital (UVH) were collected as part of routine veterinary care. The UVH human samples were collected in compliance with UVH infection control policy and approved by University College Dublin Safety, Insurance, Operational and Compliance Office i.e the samples were collected by the person themselves and were processed as screening samples only. The extra samples collected from pigs on the farm were exempt from ethical review because they were part of a clinical investigation for the farmer. No medical records or identifying information about patients or owners were accessed as part of this study. The isolates and any relevant information about the cases was obtained and analysed in a fully anonymised and de-identified form.

### Isolates

Thirty-two CC398 isolates, 22 MRSA and 10 MSSA, recovered in the ROI from animals (pigs, *n* = 17; horses, *n* = 1) and humans (*n* = 14) were investigated in the present study. The majority of isolates (*n* = 28) were identified at the UVH Microbiology Laboratory, which processes samples from UVH (a tertiary referral centre) and from private veterinary practitioners. The horse from which CC398 MRSA was isolated was one of 19 equine cases from which MRSA was isolated in the UVH Microbiology Laboratory between 2010 and 2014. CC398 MRSA was subsequently recovered from a nasal swab of the veterinarian attending the horse. The remaining UVH CC398 MRSA isolates were recovered from pigs on one farm (Farm A) or farm workers on two farms (Farms A and B) which were investigated due to the finding of CC398 MRSA in a pig from Farm A during a post mortem at UVH and an epidemiological link between Farms A and B. The porcine CC398 MSSA isolates were recovered from two farms which were investigated as part of a UVH research project where between 15% and 69% of pigs on Irish farms were MSSA positive (unpublished UVH data). CC398 MSSA represented 1.5% of MSSA isolated from one farm and 0.7% of MSSA isolated from another.

The remaining CC398 *S*. *aureus* isolates (*n* = 4) were identified at the NMRSARL between 2010 and 2014 as part of routine investigations. This represented 0.05% (2/3426) and 0.35% (2/574) of MRSA and MSSA isolates, respectively, investigated by NMRSARL, between 2010 and 2014. CC398 *S*. *aureus* represented 0.19% (4/2074) of *S*. *aureus* genotyped in the NMRSARL between 2010 and 2014. The NMRSARL investigates MRSA and MSSA isolates at the request of microbiology laboratories throughout Ireland and can include isolates recovered from various different patient and environmental sites from both hospital and community sources. It also analyses all MRSA bloodstream infection (BSI) isolates from patients in Irish hospitals that participate in The European Antimicrobial Resistance Surveillance Network (EARS-Net) project, which includes one isolate per patient per quarter from 26 participating hospitals.

Isolates were identified as *S*. *aureus* using the tube coagulase test as described previously [18] or a commercial latex agglutination assay (Pastorex Staph-Plus Bio-Rad, France). Isolates were initially assigned to CC398 by *spa* typing and this, and the species identification was confirmed by DNA microarray profiling (see below). Isolates were stored at -80°C on cryoprotective beads (Technical Service Consultants Ltd., UK).

### Antimicrobial susceptibility testing

MRSA and MSSA were differentiated using Brilliance MRSA agar (Oxoid Ltd., Basingstoke, UK) or cefoxitin disks (30-μg) (Oxoid) using the European Committee on Antimicrobial Susceptibility Testing (EUCAST) methodology and interpretive criteria [19, 20]. Isolates also underwent susceptibility testing against an additional 23 antimicrobial agents and heavy metals as described previously [21] according to EUCAST methodology [19] using previously described quality control strains, disk concentrations, and interpretive criteria [21]. In brief, where available, EUCAST disk concentrations and interpretive criteria were used [19, 21]. If not available, Clinical Laboratory Standards Institute (CLSI) disk concentrations and interpretive criteria were used, [21, 22] or for the remaining agents (including all heavy metals tested) the disk concentrations and interpretive criteria of Rossney *et al*. [21, 23] were used. The 23 agents tested were amikacin, ampicillin, cadmium acetate, chloramphenicol, ciprofloxacin, erythromycin, ethidium bromide, fusidic acid, gentamicin, kanamycin, lincomycin, mercuric chloride, mupirocin, neomycin, phenyl mercuric acetate, rifampicin, spectinomycin, streptomycin, sulphonamide, tetracycline, tobramycin, trimethoprim and vancomycin.

### Molecular characterisation of isolates

Genomic DNA for all molecular tests was extracted from all isolates by enzymatic lysis using the buffers and solutions provided with the StaphyType DNA microarray kit (Alere Technologies GmbH, Jena, Germany) and the DNeasy Blood and Tissue kit (Qiagen, Crawley, West Sussex, UK). All isolates underwent DNA microarray profiling. The DNA microarray (version 2.0) consists of a DNA microarray chip adhered to each well of a microtitre strip; each chip consists of 334 *S*. *aureus* target sequences including species-specific, antimicrobial and heavy metal resistance, SCC*mec*, virulence-associated and typing genes [24, 25]. Data generated by the StaphyType arrays were analysed for the presence or absence of these genes using Arraymate software (Alere Technologies) which can assign *S*. *aureus* isolates to sequence types (STs) and/ CCs by comparing each isolate’s DNA microarray results to those of a reference collection of previously characterised strains in the Arraymate database [25]. The DNA microarray primers, probes and protocols have been described previously in detail [24, 25].

All isolates were genotyped by *spa* typing and underwent PCRs for additional antimicrobial resistance genes commonly associated with CC398 but not included on the DNA microarray. Isolates found to harbour SCC*mec* by DNA microarray profiling underwent additional SCC*mec* typing PCRs. PCRs were performed using GoTaq Flexi DNA polymerase (Promega Corporation, Madison, Wisconsin, USA) according to the manufacturer’s instructions and a G-storm GS1 (Applied Biosystems, Foster City, CA) or a Thermo Hybaid HBPX2 (Thermo Fisher Scientific, Waltham, Massachusetts, USA) thermocycler. PCR products were visualised by conventional agarose gel electrophoresis. *spa* typing, which involves PCR and sequencing of the *S*. *aureus* protein A gene *spa* [26], was performed using the primers and thermal cycling conditions described by the European Network of Laboratories for Sequence Based Typing of Microbial Pathogens (SeqNet, www.seqnet.org.). *spa* typing PCR products were purified with the GenElute PCR clean-up kit (Sigma-Aldrich Ireland Ltd., Arklow, County Wicklow Ireland) and sequencing was performed commercially by Source Bioscience (Tramore, Waterford, Ireland) using an ABI 3730xl Sanger sequencing platform. The Ridom StaphType software version 1.3 (Ridom Gmbh, Wurzburg, Germany) was used for *spa* sequence analysis and assignment of *spa* types [27]. Antimicrobial resistance gene PCRs were performed using previously described primers and thermal cycling conditions and included detection of *spc*, *tet*(L), *erm*(T), *dfrK* and *dfrG*, [28–30]. Isolates with SCC*mec* IV were subtyped using a previously described multiplex PCR, which detects the SCC*mec* IV subtypes IVa-IVh [31]. Isolates with SCC*mec* V underwent *ccrC* allotype identification using a previously described multiplex PCR to differentiate between SCC*mec* type V (*ccrC2*) and V_T_ (*ccrC2* and *ccrC8*) [32]. Details of primers, thermal cycling conditions and control strains used are shown in S1 Table.

## Results

### Importation and zoonotic spread of multiple CC398 MRSA strains in the Republic of Ireland

The 22 CC398-MRSA isolates were from four epidemiologically distinct incidents, two of which included both human and animal isolates (Incidents 2 & 3, Table 1). All MRSA isolates were *spa* type t011 (18/22) or t034 (4/22), SCC*mec* types V_T_ (5C2 & 5 i.e. type 5 *ccr* genes (*ccrC1* allele 2), class C2 *mec* and class 5 *ccr* genes (*ccrC1* allele 8); 19/22) or IVa (2B i.e. type 2 *ccr* genes (*ccrAB2*) and class B *mec*; 3/22) and the majority lacked IEC genes (20/22) (Table 1). All isolates exhibited resistance to multiple classes of antimicrobial agents and carried multiple resistance genes including those encoding resistance to beta lactams (*blaZ* 22/22), tetracycline (*tet*(M) 22/22, *tet*(K) 18/22), *tet*(L) 6/22), macrolides, lincosamides and streptogramin B (MLS_B_) compounds (*erm*(A) 15/22, *erm*(B) 6/22, *erm*(C) 2/22), spectinomycin (*spc* 14/22), trimethoprim (*dfrG* 12/22, *dfrK* 9/22), aminoglycosides (*aacA-aphD* 5/22, *aadD* 6/22) and chloramphenicol (*fexA* 5/22) (Table 1). All isolates lacked Panton-Valentine leukocidin, enterotoxin, toxic shock toxin, exfoliative toxin and heavy metal resistance (*merA*, *merB*, *qacA* and *qacC*) genes and were susceptible to the heavy metals tested (cadmium acetate and ethidium bromide).

**Table 1:** Epidemiological, phenotypic and genotypic characteristics of CC398 methicillin-resistant and methicillin-susceptible *S*. *aureus* (MRSA and MSSA) identified in the Republic of Ireland among animals and humans.

Methicillin resistance phenotype	Incident no.	Year	No. of isolates	Host	Sample site/clinical presentation (*n*)	*spa* type ^a^	IEC type	SCC*mec* type	Antimicrobial resistance pattern^b^	Antimicrobial resistance genes
MRSA	1	2011	1	Human	Nursing home resident nasal swab	t011	Negative	IVa	Ap, Gn, Kn, Tb, Te, Tp	*blaZ*, *aacA-aphD*, *tet*(M), *dfrK*
MRSA	2	2012	2	Horse & human	Horse umbilical abscess; veterinarian nasal swab	t011	B (*sak*, *chp* & *scn*)	IVa	Ap, Er, Gn, Ln, Kn, Tb, Te, Tp	*blaZ*, *erm*(C), *aacA-aphD*, *tet*(M), *dfrG*, *dfrK*
MRSA	3	2012 & 2013	18	Pig (*n* = 9) & human (*n* = 9)	Pig joint abscess-farm A (1); pig nasal swab- farm A (1)	t034	Negative	V_T_	Ap, Er, Ln, Sp, Te, Tp	*blaZ*, *erm*(A), *tet*(K), *tet*(M), *dfrG*, *spc*
					Pig nasal swab-farm A (1)	t034	Negative	V_T_	Ap, Er, Ln, Sp, Te, Tp	*blaZ*, *erm*(A), *tet*(M), *dfrG*, *spc*
					Pig nasal swab-farm A (1); pig farm worker nasal swab-farms A (2) & B (5)	t011	Negative	V_T_	Ap, Er, Ln, Sp, Te	*blaZ*, *erm*(A), *tet*(K), *tet*(M), *spc*
					Pig farm worker nasal swab-farm A (1)	t011	Negative	V_T_	Ap, Ch, Te	*blaZ*, *tet*(K), *tet*(M), *fexA*
					Pig farm worker nasal swab-farm A (1)	t011	Negative	V_T_	Ap, Ch, Er, Kn, Ln, Nm, Tb, Te, Tp	*blaZ*, *erm*(B), *aadD*, *tet*(K), *tet*(M), *fexA*, *tet*(L), *dfrG*, *dfrK*
					Pig nasal swab-farm A (2)	t011	Negative	V_T_	Ap, Ch, Er, Kn, Ln, Gn, Nm, Tb, Te, Tp	*blaZ*, *erm*(B), *aacA-aphD*, *aadD*, *tet*(K), *tet*(M), *fexA*, *tet*(L), *dfrG*, *dfrK*
					Pig nasal swab-farm A (1)	t011	Negative	V_T_	Ap, Ch, Er, Ln, Nm, Tb, Te, Tp	*blaZ*, *erm*(A), *erm*(B), *aadD*, *tet*(M), *tet*(K) *fexA*, *tet*(L), *dfrG*, *dfrK*
					Pig nasal swab-farm A (1)	t011	Negative	V_T_	Ap, Er, Gn, Kn, Ln, Nm, Sp, Tb, Te, Tp	*blaZ*, *erm*(A), *erm*(B), *aadD*, *tet*(M), *tet*(L), *dfrG*, *dfrK*, *spc*
					Pig nasal swab-farm A (1)	t011	Negative	V_T_	Ap, Er, Kn, Ln, Nm, Sp, Te, Tb, Tp	*blaZ*, *erm*(A), *erm*(B), *aadD*, *tet*(K), *tet*(M), *dfrG*, *dfrK*, *tet*(L), *spc*
MRSA	4	2013	1	Human	Child skin abscess with family contact working with pigs	t034	Negative	V_T_	Ap, Er, Ln, Sp, Te, Tp	*blaZ*, *erm*(A), *tet*(K), *tet*(M), *dfrG*, *spc*
MSSA	5	2010	3	Pig	Nasal swabs	t108 (1)	Negative	N/A	Ap, Cp, Sp, Te	*blaZ*, *tet*(M), *spc*
						t108 (1)	Negative	N/A	Ap, Cp, Er, Ln, Sp, Te	*blaZ*, *erm*(C), *tet*(M),
						t4854 (1)	Negative	N/A	Ap, Er, Ln, Sp, Te	*blaZ*, *erm*(C), *tet*(M), *spc*
MSSA	6	2010	5	Pig	Nasal swabs	t034	Negative	N/A	Ap, Cp, Er, Ln, Sp, Te	*blaZ*, *erm*(A), *tet*(M), *spc*
MSSA	7	2014	1	Human	BSI	t571	C (*chp* & *sc*n)	N/A	Ap, Er	*blaZ*, *erm*(T)
MSSA	8	2014	1	Human	BSI	t011	D (*sea*, *sak* & *scn*)	N/A	Ap	*blaZ*

^a^*Spa* repeat successions: t011, 08-16-02-25-34-24-25; t034: 08-16-02-25-02-25-34-24-25; t108: 08-16-02-25-24-25; t571: 08-16-02-25-02-25-34-25; t4854: 08-16-02-25-24.

^b^The susceptibility of each isolate was determined against 23 antimicrobial agents including amikacin, ampicillin (Ap), cadmium acetate, chloramphenicol, ciprofloxacin (Cp), erythromycin (Er), ethidium bromide, fusidic acid, gentamicin (Gn), kanamycin (Kn), lincomycin (Ln), mercuric chloride, mupirocin, neomycin (Nm), phenyl mercuric acetate, rifampicin, spectinomycin (Sp), streptomycin, sulphonamide, tetracycline (Te), tobramycin (Tb), trimethoprim (Tp) and vancomycin.

Abbreviations: BSI, bloodstream infection; *n*, number of isolates; N/A, not applicable; IEC, immune evasion complex.

The first CC398 MRSA isolate has been reported previously [16] and was recovered in 2011 from a nursing home patient who had been a part-time cattle farmer and was *spa* type t011-SCC*mec* IVa (Incident 1, Table 1). Similar to the majority of other t011 isolates identified here, this isolate lacked IEC genes but harboured less resistance genes (Table 1). Incident 2 involved two t011-SCC*mec* IVa isolates recovered in 2012 from a horse and an attending Belgian veterinarian who had recently returned from Belgium. The veterinarian was tested for MRSA nasal carriage following identification of MRSA in the horse and was subsequently treated and successfully decolonised. Unlike Incident 1, these isolates harboured IEC genes and additional resistance determinants (*erm*(C) & *dfrG*) (Table 1).

Incident 3 yielded 18 isolates from two farms during 2012/2013. The initial farm A CC398 MRSA isolate was recovered from a pig joint abscess during a post mortem examination at UVH. Subsequently, the farm was visited and nasal swabs were collected from 100 pigs and five farm workers who had contact with the pigs. CC398 MRSA was recovered from 8/100 pigs and 4/5 farm workers sampled. This farm had been restocked prior to isolate recovery with Irish and German gilts. Farm B was a finisher unit for Farm A; all weaned pigs were transported from Farm A to Farm B at approximately 12 weeks of age. Isolates were recovered from nasal swabs of 5/10 Farm B workers. All isolates within Incident 3 harboured SCC*mec* V_T_ and lacked IEC genes. Three of the pig isolates (joint abscess and two nasal swabs) were *spa* type t034 and differed only in the absence of *tet*(K) in one nasal isolate (Table 1). Interestingly, the Incident 4 CC398-MRSA isolate, which was recovered from a child with a skin abscess, was indistinguishable in terms of *spa* and SCC*mec* type, lack of IEC and resistance gene content from 2/3 t034 pig isolates in Incident 3 (Table 1) but no epidemiological link between the isolates was identified. However, the child had worked with his father on a pig farm in the UK.

The remaining Incident 3 isolates were *spa* type t011 with seven different combinations of antimicrobial resistance genes identified (Table 1). However, one pig nasal t011-V_T_ isolate was indistinguishable from 7/9 pig farmer nasal isolates and two pig nasal t011-V_T_ isolates were indistinguishable from each other due to carriage of the same combinations of antimicrobial resistance genes detected.

### Distinct CC398 MSSA strains among animals and humans in the Republic of Ireland

Four distinct incidents involving CC398 MSSA, two from humans (BSIs, two isolates) and two from nasal carriage in pigs (eight isolates), were identified (Table 1). Isolates from animals and humans were distinguished from each other in *spa* types, IEC genes and antimicrobial resistance genes and phenotype (Table 1). The two human isolates were also distinct from each other; they were recovered from patients in two different hospitals, exhibited different *spa* types (t571 and t011) and harboured different combinations of IEC and resistance genes, with one isolate harbouring *erm*(T) (Incidents 7 and 8, Table 1). Incident 7 involved a 75-year old male and Incident 8 involved a 51-year old male but no additional information was available regarding these patients. Each pig CC398 MSSA incident consisted of multiple isolates recovered from two farms in 2010 and these isolates lacked IEC genes and harboured multiple resistance genes (Incidents 5 and 6, Table 1). Incident 5 and 6 isolates were phenotypically and genotypically distinct from each other (Table 1). Incident 5 isolates exhibited the same or closely related *spa* types and harboured similar resistance genes including *blaZ*, *tet*(M) and *spc* with 2/3 isolates also harbouring *erm*(C) and exhibiting ciprofloxacin resistance (Table 1). Incident 6 isolates exhibited a different *spa* type (t034) from Incident 5 isolates and although they harboured similar resistance genes and exhibited ciprofloxacin resistance, Incident 5 isolates harboured *erm*(A) and not *erm*(C). These t034 MSSA isolates were similar to the t034 MRSA isolates (Incident 3 & 4) but lacked *dfrG* and *tet*(K).

## Discussion

This study revealed the emergence of multiple CC398-MRSA strains among animals and humans in the ROI as well as its importation and spread and highlights a combination of inadequate biosecurity at the level of country, farm and veterinary hospital. The CC398 MRSA identified here appear to be predominantly of animal origin based on epidemiological evidence, the lack of IEC genes and the prevalence of *tet*(M) [7]. The importation of gilts from Germany, where CC398 MRSA has been reported extensively among animals and humans [33–35], to restock one of the farms and subsequent spread to other pigs and farmers on this and an additional farm highlights the ability of CC398 MRSA to spread and the introduction of novel zoonotic organisms as a consequence of open border policies. These findings have implications for both human and animal health as well as the agricultural industry in the ROI. Firstly, the risk posed by contact with livestock needs to be considered when screening high-risk groups for MRSA on admission to Irish hospitals. Furthermore, animal MRSA infections are not notifiable in the ROI and there is no requirement to screen imported animals for MRSA. There is a need to reconsider this policy and to conduct further work to establish how widely CC398 MRSA has disseminated within the Irish pig industry. While the major threat identified here is the spread of CC398 MRSA from animals to humans via direct contact, CC398 MRSA have also been reported in retail meat products including pork [13, 36], representing a further potential threat to public health and the reputation of the Irish agricultural sector. The presence of IEC in the veterinarian and horse isolates and the recent travel of the veterinarian to Belgium where IEC-positive CC398-MRSA-IV have been reported [37] suggest human to animal transmission in this instance due to inadequate infection control measures within the veterinary hospital.

The extensive antimicrobial resistance of CC398 MRSA is also of concern. As is characteristic of CC398 MRSA, all isolates identified here harboured multiple antimicrobial resistance genes encoding resistance to a range of agents used in clinical and veterinary medicine (Table 1). This multidrug resistance compromises our ability to treat CC398 MRSA infections, and due to the previously reported plasmid location of many of these resistance genes [38–40], highlights the reservoir of resistance genes that exists among LA-MRSA and the potential of these genes to spread to other *S*. *aureus* strains in animals and humans. Interestingly many of these CC398-MRSA isolates also harboured multiple genes encoding resistance to a single agent including multiple tetracycline, trimethoprim, aminoglycosides and MLS_B_ resistance genes. While this may reflect the co-location of some of these genes on a single plasmid [38] it suggests significant pressure for the selection and maintenance of these resistance genes exists, particularly among isolates from pigs and farm workers, which carried the largest number of resistance genes (Table 1).

In the present study at least four distinct CC398 MRSA strains were identified based on *spa* and SCC*mec* typing and detection of IEC genes including (i) t011-IVa, IEC negative, (ii) t011-IVa, IEC positive, (iii) t034-V_T_, IEC negative, (iv) t011-V_T_, IEC negative, (Table 1). Within the t034- and t011-V_T_ isolates differences were detected in the combinations of antimicrobial resistance genes that they harboured. The three farm A and B t034-V_T_ isolates differed only in the absence of *tet*(K) in one isolate and two of these were indistinguishable from the child skin abscess t034-V_T_ isolate in terms of the antimicrobial resistance genes detected suggesting the possible spread of a single strain. However, this child had worked with his father on a pig farm in the UK, indicating that the CC398 MRSA infection may have been acquired through contact with pigs in the UK. Similar to the ROI, it is not known how widespread CC398 MRSA is among pigs in the UK with just three piglets reported to date with CC398 MRSA, two of which were assigned to the same *spa* type (t034) and a similar SCC*mec* type (V) to the child skin abscess CC398 MRSA isolate in the present study [11, 12]. The 15 farm A and B t011-V_T_ isolates harboured between four and 10 resistance genes each and were differentiated into seven groups based on the different combinations of these genes (Table 1). While the differences detected in resistance gene content may indicate the presence of multiple distinct t011-V_T_ CC398 MRSA strains, these differences may also represent the loss and gain of plasmids encoding resistance genes due to different selective pressures. Further studies using whole-genome sequencing are required to determine the precise relationship between these CC398 MRSA isolates with the same *spa* and SCC*mec* types but harbouring different combinations of antimicrobial resistance genes.

Based on molecular epidemiological typing, the animal and human CC398 MSSA isolates identified here were unrelated indicating their independent emergence and both harboured traits typical of animal and human CC398 MSSA clades, respectively [4, 5, 7] i.e. the human isolates were IEC positive and carried only one or two resistance genes with one harbouring *erm*(T) while the pig isolates were IEC negative and harboured multiple resistance genes. Just two human CC398 MSSA isolates, both from BSIs, were identified, in 2014. A low but increasing level of CC398 MSSA among human invasive infections have been reported elsewhere in Europe [5, 41]. While molecular epidemiological typing did not reveal the spread of CC398 MSSA from animals to humans or *vice versa*, it did reveal the spread of CC398 MSSA among pigs on two farms. Two of the *spa* types identified among the CC398 MSSA were also reported among the CC398 MRSA (t034 and t011) and these may be potential precursors for the emergence of CC398 MRSA. A recent study highlighted the presence of SCC*mec* remnants in CC398 MSSA and suggested that CC398 MRSA could emerge from these [6]. However no SCC*mec* genes were identified among the CC398 isolates identified here.

In conclusion, this study has, for the first time, revealed the importation and zoonotic spread of multiple multidrug resistant CC398 MRSA strains in the ROI and the spread of CC398 MSSA among pigs. It has also highlighted the reservoir of resistance genes that exists among CC398 MRSA that could potentially spread to other animal and human *S*. *aureus* strains. Increased surveillance of humans and animals in the ROI is warranted to prevent further CC398 MRSA importation and spread in a country that was, until recently, considered CC398 MRSA free.

## Supporting Information

S1 Table Details of primers, thermal cycling conditions and positive control strains used in the present study. (PDF)
