# Supplementary material for: The Emergence and Spread of Multiple Livestock-Associated Clonal Complex 398 Methicillin-Resistant and Methicillin-Susceptible Staphylococcus aureus Strains among Animals and Humans in the Republic of Ireland, 2010–2014
Source: PLoS One. 2016 Feb 17;11(2):e0149396. doi: 10.1371/journal.pone.0149396 (PMC4757405; doi:10.1371/journal.pone.0149396)
Supplement: S1 Table — (PDF) [file pone.0149396.s001.pdf]

Supplemental S1 Table. Details of primers, thermal cycling conditions and positive control strains used in the present study

Primer use	Gene region/target	Primer name	Primer sequence 5' - 3'	Expected amplimer size (bp)	Thermal cycling conditions	Reference	Positive control strain [Reference]
***spa* typing**	*spa*	spa-1113f<br>spa-1514r	AGACGATCCTTCGGTGAGC<br>AGACGATCCTTCGGTGAGC	200-600	80°C for 5 min; 35 cycles of 94°C for 45 s, 60°C for 45 s and 72°C for 90 s, and a final extension at 72° C for 10 min	www.seqnet.org.	None used
**SCC*mec* IV subtyping multiplex PCR**	IVa	J IVa F<br>J IVa R	ATAAGAGATCGAACAGAAGC<br>TGAAGAAATCATGCCTATCG	278	94°C for 4 min; 35 cycles of 94°C for 30 s, 48°C for 30 s and 72°C for 2 min and a final extension at 72°C for 4 min	[1]	MRSA CA05 [2]
	IVb/IVF	J IVb F<br>J IVb R	TTGCTCATTTCAGTCTTACC<br>TTACTTCAGCTGCATTAAGC	336			MRSA 8/63P [2]
	IVc/IVE	J IVc F<br>J IVc R	CCATTGCAAATTTCTCTTCC<br>ATAGATTCTACTGCAAGTCC	483			MRSA JCSC4788 [3]
	IVd	J IVd F<br>J IVd R	TCTCGACTGTTTGCAATAGG<br>CAATCATCTAGTTGGATACG	575			MRSA JCSC4469 [3]
	IVg	J IVg F<br>J IVg R	TGATAGTCAAAGTATGGTGG<br>GAATAATGCAAAGTGGAACG	792			MRSA M04/0177 [4]
	IVh	J IVh F<br>J IVh R	TTCCTCGTTTTTTCTGAACG<br>CAAACACTGATATTGTGTCG	663			MRSA E1749 [4]
	ccrB2[a]	ccrB2 F<br>ccrB2 R	CGAACGTAATAACATTGTCG<br>TTGGCWATTTTACGATAGCC	203			MRSA CA05 [2]
**SCC*mec* V subtyping multiplex PCR**	*ccrC2*	ccrC2-F2<br>ccrC2-R2	ATAAGTTAAAAGCACGACTCA<br>TTCAATCCTATTTTTCTTTGTG	257	95°C for 2 min; 35 cycles of 95°C for 1 min, 55°C for 30 s and 72°C for 30 s and a final extension at 72°C for 2 min	[5]	MRSA M06/0318 [4]
	ccrC8	ccrC8-F<br>ccrC8-R	GCATGGGTACTCAATCCA<br>GGTTGTAATGGCTTTGAGG	562			MRSA M06/0318 [4]

**PCR amplification of additional resistance genes**	*spc*	spc_fw<br>spc_rv	ACCAAATCAAGCGATTCAAA<br>GTCACTGTTTGCCACATTCG	561	94°C for 2 min; 30 cycles of 94°C for 1 min, 52°C for 1 min and 72°C for 1 min and a final extension at 72°C for 5 min	[6]	MRSA M13/0699 [this study]
	tet(L)	tetL-F<br>tetL-R	TCGTTAGCGTGCTGTCATTC<br>GTATCCCACCAATGTAGCCG	267	94°C for 2 min; 30 cycles of 94°C for 1 min, 55°C for 30 sec and 72°C for 1 min and a final extension at 72°C for 5 min	[7]	*Escherichia coli* pB*2187dfrK* [8]
	dfrK	dfrK_fw<br>dfrK_rv	CAAGAGATAAGGGGTTCAGC<br>ACAGATACTTCGTTCCACTC	229	94°C for 2 min; 30 cycles of 94°C for 1 min, 55°C for 30 sec and 72°C for 1 min and a final extension at 72°C for 5 min	[9]	*Escherichia coli* pB*2187dfrK* [8]
	dfrG	dfrG-1<br>dfrG-2	TGCTGCGATGGATAAGAA<br>TGGGCAAATACCTCATTCC	405	94°C for 2 min; 30 cycles of 94°C for 1 min, 55°C for 30 sec and 72°C for 1 min and a final extension at 72°C for 5 min	[9]	MSSA CM.S2 [10]
	erm(T)	ermT_fw<br>ermT_rv	ATTGGTTCAGGGAAAGGTCA<br>GCTTGATAAAATTGGTTTTTGGA	536	94°C for 2 min; 30 cycles of 94°C for 1 min, 45°C for 1 min and 72°C for 1 min and a final extension at 72°C for 5 min	[6]	MSSA RN4220pKKS2 5 [11]

[a] *ccrAB2* was used an internal positive control for SCC*mec* IV subtyping PCRs.

REFERENCES

1. Milheirico C, Oliveira DC, de Lencastre H. Multiplex PCR strategy for subtyping the staphylococcal cassette chromosome *mec* type IV in methicillin-resistant *Staphylococcus aureus*: 'SCC*mec* IV multiplex'. J. Antimicrob. Chemother. 2007;60(1):42-48.

2. Ma XX, Ito T, Tiensasitorn C, Jamklang M, Chongtrakool P, Boyle-Vavra S, et al. Novel type of staphylococcal cassette chromosome mec identified in community-acquired methicillin-resistant *Staphylococcus aureus* strains. Antimicrob. Agents Chemother. 2002;46(4):1147-1152.

3. Ma XX, Ito T, Chongtrakool P, Hiramatsu K. Predominance of clones carrying Panton-Valentine leukocidin genes among methicillin-resistant *Staphylococcus aureus* strains isolated in Japanese hospitals from 1979 to 1985. J. Clin. Microbiol. 2006;44(12):4515-4527.

4. Kinnevey PM, Shore AC, Brennan GI, Sullivan DJ, Ehricht R, Monecke S, et al. Extensive genetic diversity identified among sporadic methicillin-resistant *Staphylococcus aureus* isolates recovered in Irish hospitals between 2000 and 2012. Antimicrob. Agents Chemother. 2014;58(4):1907-1917.

5. Higuchi W, Takano T, Teng LJ, Yamamoto T. Structure and specific detection of staphylococcal cassette chromosome *mec* type VII. Biochem. Biophy. Res. Commun. 2008;377(3):752-756.

6. Fessler A, Scott C, Kadlec K, Ehricht R, Monecke S, Schwarz S. Characterization of methicillin-resistant *Staphylococcus aureus* ST398 from cases of bovine mastitis. J. Antimicrob. Chemother. 2010;65(4):619-625.

7. Ng LK, Martin I, Alfa M, Mulvey M. Multiplex PCR for the detection of tetracycline resistant genes. Mol. Cell. Probes. 2001;15(4):209-215.

8. Kadlec K, Schwarz S. Identification of a novel trimethoprim resistance gene, *dfrK*, in a methicillin-resistant *Staphylococcus aureus* ST398 strain and its physical linkage to the tetracycline resistance gene *tet*(L). Antimicrob. Agents Chemother. 2009;53(2):776-778.

9. Argudin MA, Tenhagen BA, Fetsch A, Sachsenroder J, Kasbohrer A, Schroeter A, et al. Virulence and resistance determinants of German *Staphylococcus aureus* ST398 isolates from nonhuman sources. Appl. Environ. Microbiol. 2011;77(9):3052-3060.

10. Sekiguchi J, Tharavichitkul P, Miyoshi-Akiyama T, Chupia V, Fujino T, Araake M, et al. Cloning and characterization of a novel trimethoprim-resistant dihydrofolate reductase from a nosocomial isolate of *Staphylococcus aureus* CM.S2 (IMCJ1454). Antimicrob. Agents Chemother. 2005;49(9):3948-3951.

11. Kadlec K, Schwarz S. Identification of a Plasmid-Borne Resistance Gene Cluster Comprising the Resistance Genes *erm*(T), *dfr*K, and *tet*(L) in a Porcine Methicillin-Resistant *Staphylococcus aureus* ST398 Strain. Antimicrob. Agents Chemother. 2010;54(1):915-918.
